# 74. Evaluation of the Differences in Appropriateness of BioFire® FilmArray® Gastrointestinal Panel Testing between Emergency Department and Inpatient Services

**DOI:** 10.1093/ofid/ofab466.276

**Published:** 2021-12-04

**Authors:** Brittani Weichman, Amanda Bushman, Rossana M Rosa

**Affiliations:** 1 UnityPoint Des Moines Iowa Lutheran Hospital, Des Moines, IA; 2 UnityPoint Health, Des Moines, IA

## Abstract

**Background:**

Use of rapid molecular diagnostic panels in the evaluation of diarrhea provides increased sensitivity for organism identification and decreased time to results. However, their inappropriate use can lead to unnecessary expenditures and antimicrobial exposures. We aimed to characterize the appropriateness of testing using the BioFire® FilmArray® Gastrointestinal Panel (SFA) in different clinical settings and to describe the impact of SFA results on patient care.

**Methods:**

Retrospective study of adult patients presenting to hospitals part of an integrated health system in Des Moines, Iowa, between July 30 and September 30, 2019, and who had a SFA ordered and collected in the Emergency Department (ED) or an inpatient service. The appropriateness of SFA testing was determined according to adherence to a local algorithm available through the hospital’s intranet (Figure 1). Reason for testing, appropriateness of SFA test, molecular targets identified, and antibiotic exposures were collected.

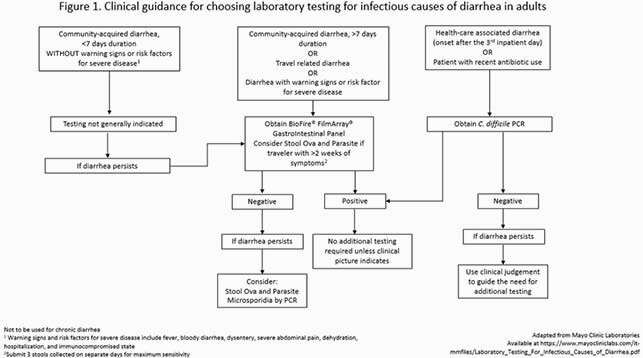

**Results:**

We identified 257 patients, 111 (43.2%) who had SFA done in the ED and 146 (56.8%) as inpatients. Testing was deemed inappropriate in 46 (41.4%) of ED patients compared to 100 (68.5%) of inpatients (*p*< 0.0001). Documented indications for SFA are presented in Table 1. Among ED patients testing was most frequently considered inappropriate due to absence of diarrhea on the day of test collection (41.3%), and among inpatients due to the use of SFA for assessment of hospital-onset diarrhea (47.0%) (Table 2). Overall, there were 94 (36.6%) positive SFA (Figure 2). Among ED patients, the percentage of positive SFA samples was 30.4% and 50.8% for inappropriate and appropriate testing respectively (*p=*0.03), while for inpatients it was 33.0% for inappropriate orders and 30.4% for appropriate orders (*p*=0.76). Antibiotics were prescribed to 28.2% and 28.1% of patients tested in the ED and inpatient service respectively.

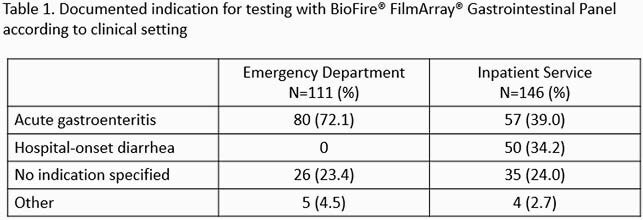

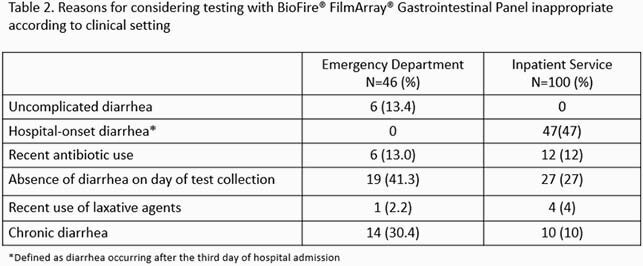

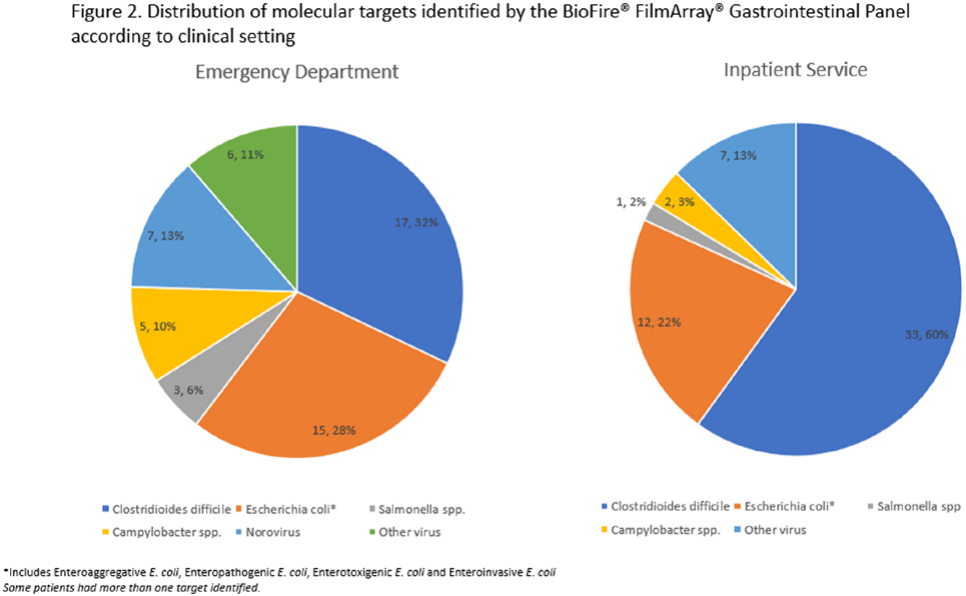

**Conclusion:**

High proportions of inappropriate SFA testing were identified both in the ED and inpatient services, with distinct issues in each site. Characterization of the reasons underlying inappropriate use of SFA can aid in the design of diagnostic stewardship interventions tailored to each clinical setting.

**Disclosures:**

**All Authors**: No reported disclosures

